# Supplementary material for: Association between chronotype and dual-task gait cost across distinct cognitive domains in healthy young adults
Source: PLoS One. 2026 Aug 31;21(8):e0348387. doi: 10.1371/journal.pone.0348387 (PMC13529012; doi:10.1371/journal.pone.0348387)
Supplement: Supplementary file 1 [file pone.0348387.s001.pdf]

ScreenIT Report

This is an automated report for 10.64898+2026.04.16.719112.pdf. This report was solicited by the PLOS One editorial team and provided by ScreenIT.

ScreenIT is an independent group of scientists developing automated tools that analyze academic papers. A set of automated tools screened your submitted manuscript and provided the report below. Each tool was created by your academic colleagues with the goal of helping authors.

The tools look for factors that are important for transparency, rigor and reproducibility, and we hope that the report might help you to improve reporting in your manuscript. Within the report you will find links to more information about the items that the tools check. These links include helpful papers, websites, or videos that explain why the item is important.

While our screening tools aim to improve and maintain quality standards they may, on occasion, miss nuances specific to your study type or flag something incorrectly. Each tool has limitations that are described on the ScreenIT website. The tools screen the main file for the paper; they are not able to screen supplements stored in separate files.

Please note that the Academic Editor had access to these comments while making a decision on your manuscript. The Academic Editor may ask that issues flagged in this report be addressed.

If you would like to provide feedback on the ScreenIT tool, please email the team at ScreenIt@bih-charite.de. If you have questions or concerns about the review process, please contact the PLOS One office at plosone@plos.org.

How to read the report:

- no action required
- review for potential issues
- a critical issue was found by a tool
- may not be applicable to your paper

- more information about this item can be obtained using this link

Rigor

n/a	Ethics	not required.
✓	Inclusion and Exclusion Criteria	"Inclusion criteria were: age 18 years; ability to walk independently without an assistive device; and English fluency sufficient to follow verbal instructions and complete self-report questionnaires."
✓	Flow charts and attrition	Thank you for including a study flow chart and information about attrition and exclusions to help readers evaluate the risk of bias.<br><br>Sentence about attrition: "(n = 70 assessed; n = 69 included)."
✓	Sex as a biological variable	"Flowchart of participants recruitmentResults Participant characteristics A total of 69 participants (37 females, 32 males) with a mean age of 21.28 2.83 years (range 1835) were included."
✓	Subject Demographics	Age: "Inclusion criteria were: age 18 years; ability to walk independently without an assistive device; and English fluency sufficient to follow verbal instructions and complete self-report questionnaires."
?	Randomization	Not detected. If you performed an experimental study, please specify whether participants, animals or samples were randomly assigned to treatments or groups, and specify the randomization procedure.
?	Blinding	Not detected. Please specify whether blinding was used at various phases of the experiment (e.g., blinding of patients, caregivers, outcome assessments, data analysis).
?	Power Analysis	Not detected. Please state how you determined the sample size for the study. If you performed a power or sample size calculation, please provide details.
✓	Replication	"This pattern was replicated across all eight gait parameters, as shown in Table 2."

Generated by Barzooka (flow charts), SciScore (everything else).
SciScore only screens the text of the methods section; we apologize if the tool has missed items reported elsewhere in the paper or supplements.

Resources

No key resources detected.
Generated by SciScore. SciScore only screens the text of the methods section; we apologize if the tool has missed items reported elsewhere in the paper or supplements.

Transparency

?	Open code	If you wrote code to analyze your data, please consider sharing the code in a public repository. This makes it easier for others to reproduce your analyses, and may also aid others seeking to analyze similar datasets.
?	Open data	We found one or more references to open data in your manuscript that did not seem to be included in your data availability statement: " data availability all data supporting the findings of this study are publicly available on zenodo: https://doi.org/10.5281/zenodo.17878983 table 1.".If these are references to your own data, make sure to include them in the data availability statement. If these are data citations, make sure to clearly state that you used previously published data.
✓	Self-acknowledged limitations	"This limitation represents the primary constraint on interpretation of the present findings and precludes definitive conclusions regarding chronotype-specific mechanisms, particularly given that higher-order cognitive functions mediated by prefrontal networks are known to be sensitive to circadian modulation [8]. Methodological considerations and limitations Several limitations should be considered when interpreting these findings. First, and most critically, all assessments were conducted at a fixed time of day without alignment to participants' individual circadian preferences. This design confounds chronotype with time-ofday effects and is the primary constraint on causal interpretation, as discussed above. Despite these limitations, the present findings provide evidence that chronotype is associated with dual-task gait performance in a cognitively specific manner, rather than as a

		general effect across all task types. This contributes to understanding of individual variability in c..."
?	Conflict of interest statement	It is important to supply a conflict of interest statement, even if there are none to declare, to transparently present the research.
?	Funding statement	It is important to supply a funding statement because it makes the funding source and potential conflicts of interests transparent.
n/a	Registration statement	A registered protocol makes the planned research project more transparent and can protect against bias. Please consider registering future confirmatory studies.

Generated by ODDPub, limitation-recognizer, rtransparent, trial-identifier.

Figures

✓	No bar graphs of continuous data	Thank you for not using bar graphs to present continuous data. Many different datasets and data distributions can lead to the same bar graph, and the actual data may suggest different conclusions from the summary statistics alone.
✓	No rainbow color maps	Thank you for not using rainbow colormaps. Rainbow color maps distort readers' perception of the data by introducing visual artifacts and are not accessible to colorblind readers.

Generated by JetFighter, Barzooka.
