# Supplementary material for: Association between chronotype and dual-task gait cost across distinct cognitive domains in healthy young adults
Source: PLoS One. 2026 Aug 31;21(8):e0348387. doi: 10.1371/journal.pone.0348387 (PMC13529012; doi:10.1371/journal.pone.0348387)
Supplement: Supplementary file 2 [file pone.0348387.s003.docx]

**Response to Reviewers**

Manuscript ID: PONE-D-26-18506

*Title: Association between chronotype and dual-task gait cost across distinct cognitive domains in healthy young adults*

We thank the editors and reviewers for their careful reading of our manuscript and for the constructive comments provided. We have revised the manuscript accordingly. All changes are visible as tracked insertions in the revised document. Below we address each point raised by Reviewer #1 in turn. Reviewer #2 comprised an automated ScreenIT report; we have reviewed all flagged items and addressed these separately below.

**Reviewer #1**

**Comment 1: Sample restricted to healthy young adults**

*"By limiting the participants to healthy young adults, the research misses the chance to explore how these relationships manifest in older individuals or those with health conditions, who often struggle most with these simultaneous demands."*

**Response:** We agree that this is a significant limitation. We included an explicit declaration in the Limitations section that the sample was limited to healthy young adults and that generalizability to older persons and clinical populations cannot be assumed. We have also included this for future research in the Implications and Future Directions section, explicitly urging for enrollment of older persons and clinical populations who experience the most functional consequences of impaired dual-task walking. We would like to clarify that the scope of the current study was deliberately limited to healthy young adults as a necessary first step in establishing baseline chronotype-gait associations before expanding to more complex populations where additional confounders such as medication, pathology, and age-related motor impairments would need to be addressed.

**Change in manuscript:** New tracked insertion in Limitations and expansion of Future Directions item (5).

**Comment 2: Single afternoon session and time-of-day confound**

*"Conducting all assessments during a single afternoon session makes it difficult to ascertain whether the results truly stem from an individual’s internal rhythm or if they are merely a snapshot of mid-day energy levels. Without comparing these results against different times of day, such as early morning or late evening, the core argument regarding the influence of our biological clock remains somewhat incomplete."*

**Response:** We completely agree that this is the most important drawback of the current study, and we addressed it openly in both the Discussion and Limitations sections of the original paper. In the revised version, we strengthened this acknowledgement with a tracked insertion in the Limitations which presently explicitly calls for within-participant repeated measurement designs assessing each participant at various times across the day to isolate chronotype trait effects from temporary time-of-day arousal fluctuations and directly test the synchrony hypothesis. We recognize that the current cross-sectional single-session design restricts causal inference, and we have presented all data as associations compatible with the synchronization hypothesis rather than verified circadian mechanisms throughout the text.

**Change in manuscript:** Tracked insertion expanding Limitation; comment marker added to Study Design section.

**Comment 3: Absence of cognitive task accuracy data**

*"The primary focus on walking mechanics at the expense of mental performance. Without knowing how accurately the participants performed the mental tasks — whether they were solving the problems correctly or simply guessing — it is hard to determine if they were shifting their effort from thinking to walking."*

**Response:** This is a valid methodological concern. The assessor verbally assessed cognitive task accuracy and reaction rates to ensure continued task engagement, but no formal scores were recorded on a trial-by-trial basis. In the revised manuscript, we added a tracked insertion in the Limitations section, emphasizing that without accuracy data, it is challenging to determine whether participants were successfully completing the cognitive tasks or randomly responding, which would conceal genuine cognitive-motor interference. We noted that the assessor constantly evaluated verbal engagement. during each trial, which gives proof that individuals were actively performing the tasks rather than disengaging completely.

**Change in manuscript:** Tracked insertion expanding Limitations; comment marker added to Dual-task conditions paragraph in Methods.

**Comment 4: Laboratory setting and ecological validity**

*"The laboratory setting, while useful for control, lacks the unpredictability of a real-world environment where distractions and uneven surfaces are the norm."*

**Response:** We agree. A new limitation paragraph has been added stating that it still needs to be determined if the observed chronotype-DTC associations would replicate under ecologically representative conditions. Mainly, we would like to point out that the controlled laboratory environment was a conscious design choice that allowed for exact spatiotemporal gait assessment using a three-dimensional motion capture device, which would not be possible in uncontrolled outdoor situations. However, we have expressly proposed the use of wearable inertial measurement devices deployed in realistic community settings as a viable next step, and ecological validity testing has been included under implications and future directions.

**Change in manuscript:** New tracked insertion as Limitation; Future Directions expanded with item (6).

**Comment 5: Absence of data on daily variables**

*"The absence of data on daily variables like sleep quality from the night before, stress levels, or caffeine consumption leaves several external factors unaccounted for, all of which could significantly sway both mental agility and the rhythm of a person’s stride."*

**Response:** We thank the reviewer for bringing this up. We would want to highlight that all participants' daytime drowsiness was assessed before to testing using the Epworth drowsiness Scale (ESS), a validated tool for detecting clinically relevant levels of daily sleepiness. Participants with ESS scores suggesting extreme daytime drowsiness were excluded from the research. This screening step immediately addresses the worry that poor prior-night sleep quality may have severely affected cognitive or motor performance on the day of testing, as participants whose sleep quality was poor enough to cause observable daytime drowsiness were excluded from the analysis.

We accept that the ESS measures habitual daytime drowsiness rather than acute same-day sleep quality, and that additional daily-state factors, such as morning coffee consumption and subjective stress levels on the testing day, were not systematically examined. These variables may impact both cognitive arousal and gait regularity, regardless of chronotype. We have included a paragraph in the limitations to acknowledge that and to state the need for future studies using structured day-of-testing questionnaires including, such as a single-night sleep quality item, a caffeine intake log, and a brief subjective stress rating, to separate acute daily-state effects from the stable chronotype trait. This recommendation is also listed under the future directions section.

**Change in manuscript:** Tracked insertion in Limitations; Future Directions expanded with item (7).

**Reviewer #2 – ScreenIT Automated Report**

**Item 1: Randomisation not detected**

*"Not detected. If you performed an experimental study, please specify whether participants, animals or samples were randomly assigned to treatments or groups, and specify the randomisation procedure."*

**Response:** this study is an observational cross-sectional study that does not involve an experimental protocol. The aim of this study was to evaluate the association between chronotype and dual-task cost on gait during various dual-tasks. Thus, randomization was not required. However, participants were recruited using convenience sampling.

**Item 2: Blinding not detected**

*"Not detected. Please specify whether blinding was used at various phases of the experiment (e.g., blinding of patients, caregivers, outcome assessments, data analysis)."*

**Response:** the following paragraph has been added in the manuscript to address blinding: considering the nature of the dual-task conditions, complete blinding of participants to the dedicated task was not applicable. However, to minimize demand characteristics, participants were not informed about the study hypotheses regarding the association between chronotype and gait performance. Moreover, the assessor who recorded gait data was not engaged in MEQ scoring or chronotype classification of participants, thus providing partial blinding for the primary outcome measure for assessors.

**Item 3: Power analysis not detected**

*"Not detected. Please state how you determined the sample size for the study. If you performed a power or sample size calculation, please provide details."*

**Response:** A new subsection titled “Sample size” has been added and the following paragraph has been added to address sample size determination: Sample size determination was performed using G*Power (version 3.1). Assuming a medium-to-large effect size (f² = 0.20) based on previous chronotype–cognition research, α = 0.05, power = 0.80, and five predictors (MEQ score, age, sex, BMI, and baseline gait performance), a minimum sample size of 55 participants was required.

**Item 4: Open code**

*"If you wrote code to analyse your data, please consider sharing the code in a public repository. This makes it easier for others to reproduce your analyses and may also aid others seeking to analyse similar datasets."*

**Response:** All statistical analyses were performed using IBM SPSS Statistics (version 25) via its graphical interface. No custom analysis code was written; therefore code sharing is not applicable to this study. All underlying data are publicly available on Zenodo (https://doi.org/10.5281/zenodo.17878983).

**Item 5: Open data**

*"We found one or more references to open data in your manuscript that did not seem to be included in your data availability statement."*

**Response:** The Zenodo link refers to our own primary dataset. The Data Availability statement in the revised manuscript has been expanded to explicitly confirm that all individual-level data underlying the findings, including spatiotemporal gait parameters, dual-task cost values, MEQ scores, ESS scores, SOMCT scores, and demographic variables, are publicly available at https://doi.org/10.5281/zenodo.17878983, with no data withheld.

**Item 6: Conflict of interest statement**

*"It is important to supply a conflict-of-interest statement, even if there are none to declare, to transparently present the research."*

**Response:** The authors declared no conflict of interest. A clear conflict of interest statement has been added at the end of the manuscript.

**Item 7: Funding statement**

*"It is important to supply a funding statement because it makes the funding source and potential conflicts of interest transparent."*

**Response:** This research received no external funding. A funding statement confirming this has been added to the revised manuscript.
