# Supplementary material for: Association between chronotype and dual-task gait cost across distinct cognitive domains in healthy young adults
Source: PLoS One. 2026 Aug 31;21(8):e0348387. doi: 10.1371/journal.pone.0348387 (PMC13529012; doi:10.1371/journal.pone.0348387)
Supplement: Peer review history

# Peer review: Decision Letter 0

**DOI:** 10.1371/journal.pone.0348387.r001

**Authors:** Julio Alejandro Henriques da Costa

28 May 2026

Dear Dr. Kim,

Thank you for submitting your manuscript to PLOS ONE. After careful consideration, we feel that it has merit but does not fully meet PLOS ONE’s publication criteria as it currently stands. Therefore, we invite you to submit a revised version of the manuscript that addresses the points raised during the review process.

Please submit your revised manuscript by Jul 12 2026 11:59PM. If you will need more time than this to complete your revisions, please reply to this message or contact the journal office at plosone@plos.org. When you're ready to submit your revision, log on to https://www.editorialmanager.com/pone/ and select the 'Submissions Needing Revision' folder to locate your manuscript file.

- A letter that responds to each point raised by the academic editor and reviewer(s). You should upload this letter as a separate file labeled 'Response to Reviewers'.
- A marked-up copy of your manuscript that highlights changes made to the original version. You should upload this as a separate file labeled 'Revised Manuscript with Track Changes'.
- An unmarked version of your revised paper without tracked changes. You should upload this as a separate file labeled 'Manuscript'.

If you would like to make changes to your financial disclosure, please include your updated statement in your cover letter. Guidelines for resubmitting your figure files are available below the reviewer comments at the end of this letter.

If applicable, we recommend that you deposit your laboratory protocols in protocols.io to enhance the reproducibility of your results. Protocols.io assigns your protocol its own identifier (DOI) so that it can be cited independently in the future. For instructions see: https://journals.plos.org/plosone/s/submission-guidelines#loc-laboratory-protocols. Additionally, PLOS ONE offers an option for publishing peer-reviewed Lab Protocol articles, which describe protocols hosted on protocols.io. Read more information on sharing protocols at https://plos.org/protocols?utm_medium=editorial-email&utm_source=authorletters&utm_campaign=protocols.

As the corresponding author, your ORCID iD is verified in the submission system and will appear in the published article. PLOS supports the use of ORCID, and we encourage all coauthors to register for an ORCID iD and use it as well. Please encourage your coauthors to verify their ORCID iD within the submission system before final acceptance, as unverified ORCID iDs will not appear in the published article. *Only*  the individual author can complete the verification step; PLOS staff *cannot*  verify ORCID iDs on behalf of authors.

We look forward to receiving your revised manuscript.

Kind regards,

Julio Alejandro Henriques Castro da Costa

Academic Editor

PLOS One

Journal Requirements:

When submitting your revision, we need you to address these additional requirements.

1. Please ensure that your manuscript meets PLOS ONE's style requirements, including those for file naming. The PLOS ONE style templates can be found at https://journals.plos.org/plosone/s/file?id=wjVg/PLOSOne_formatting_sample_main_body.pdf and https://journals.plos.org/plosone/s/file?id=ba62/PLOSOne_formatting_sample_title_authors_affiliations.pdf

2. Thank you for stating the following in your Competing Interests section:

[no competing interests].

Please complete your Competing Interests on the online submission form to state any Competing Interests. If you have no competing interests, please state "The authors have declared that no competing interests exist.", as detailed online in our guide for authors at http://journals.plos.org/plosone/s/submit-now

This information should be included in your cover letter; we will change the online submission form on your behalf.

3. Please include captions for your Supporting Information files at the end of your manuscript, and update any in-text citations to match accordingly. Please see our Supporting Information guidelines for more information: http://journals.plos.org/plosone/s/supporting-information.

If the reviewer comments include a recommendation to cite specific previously published works, please review and evaluate these publications to determine whether they are relevant and should be cited. There is no requirement to cite these works unless the editor has indicated otherwise.

[Note: HTML markup is below. Please do not edit.]

Reviewers' comments:

Reviewer's Responses to Questions

**Comments to the Author**

1. Is the manuscript technically sound, and do the data support the conclusions?

Reviewer #1: Partly

Reviewer #2: Yes

**********

2. Has the statistical analysis been performed appropriately and rigorously?

Reviewer #1: I Don't Know

Reviewer #2: Yes

**********

3. Have the authors made all data underlying the findings in their manuscript fully available?

The PLOS Data policy

Reviewer #1: No

Reviewer #2: Yes

**********

4. Is the manuscript presented in an intelligible fashion and written in standard English?

Reviewer #1: No

Reviewer #2: Yes

**********

Reviewer #1: In reviewing this manuscript, I appreciate the effort to bridge the gap between our internal biological clocks and the daily challenge of balancing movement with mental focus. However, there are several areas where the study could have delved deeper to provide a more holistic understanding. By limiting the participants to healthy young adults, the research misses the chance to explore how these relationships manifest in older individuals or those with health conditions, who often struggle most with these simultaneous demands. Additionally, conducting all assessments during a single afternoon session makes it difficult to ascertain whether the results truly stem from an individual’s internal rhythm or if they are merely a snapshot of mid-day energy levels. Without comparing these results against different times of day, such as early morning or late evening, the core argument regarding the influence of our biological clock remains somewhat incomplete.

Another point to consider is the primary focus on walking mechanics at the expense of mental performance. Without knowing how accurately the participants performed the mental tasks whether they were solving the problems correctly or simply guessing it is hard to determine if they were shifting their effort from thinking to walking. Furthermore, the laboratory setting, while useful for control, lacks the unpredictability of a real-world environment where distractions and uneven surfaces are the norm. Lastly, the absence of data on daily variables like sleep quality from the night before, stress levels, or caffeine consumption leaves several external factors unaccounted for, all of which could significantly sway both mental agility and the rhythm of a person’s stride.

Reviewer #2: This is an automated report for PONE-D-26-18506. This report was solicited by the PLOS One editorial team and provided by ScreenIT.

ScreenIT is an independent group of scientists developing automated tools that analyze academic papers. A set of automated tools screened your submitted manuscript and provided the report below. Each tool was created by your academic colleagues with the goal of helping authors. The tools look for factors that are important for transparency, rigor and reproducibility, and we hope that the report might help you to improve reporting in your manuscript. Within the report you will find links to more information about the items that the tools check. These links include helpful papers, websites, or videos that explain why the item is important. While our screening tools aim to improve and maintain quality standards they may, on occasion, miss nuances specific to your study type or flag something incorrectly. Each tool has limitations that are described on the ScreenIT website. The tools screen the main file for the paper; they are not able to screen supplements stored in separate files. Please note that the Academic Editor had access to these comments while making a decision on your manuscript. The Academic Editor may ask that issues flagged in this report be addressed. If you would like to provide feedback on the ScreenIT tool, please email the team at ScreenIt@bih-charite.de. If you have questions or concerns about the review process, please contact the PLOS One office at plosone@plos.org.

**********

what does this mean?). If published, this will include your full peer review and any attached files.

If you choose “no”, your identity will remain anonymous but your review may still be made public.

**Do you want your identity to be public for this peer review?**  For information about this choice, including consent withdrawal, please see our Privacy Policy

Reviewer #1: No

Reviewer #2: No

**********

[NOTE: If reviewer comments were submitted as an attachment file, they will be attached to this email and accessible via the submission site. Please log into your account, locate the manuscript record, and check for the action link "View Attachments". If this link does not appear, there are no attachment files.]

To ensure your figures meet our technical requirements, please review our figure guidelines: https://journals.plos.org/plosone/s/figures

You may also use PLOS’s free figure tool, NAAS, to help you prepare publication quality figures: https://journals.plos.org/plosone/s/figures#loc-tools-for-figure-preparation.

NAAS will assess whether your figures meet our technical requirements by comparing each figure against our figure specifications.

Attachment Submitted filename: report_10.64898+2026.04.16.719112.pdf

# Author comment: Author response to Decision Letter 1

**DOI:** 10.1371/journal.pone.0348387.r002

18 Jul 2026

Response to Reviewers has been uploaded as a separate file. Below is the same content.

Manuscript ID: PONE-D-26-18506

Title: Association between chronotype and dual-task gait cost across distinct cognitive domains in healthy young adults

We thank the editors and reviewers for their careful reading of our manuscript and for the constructive comments provided. We have revised the manuscript accordingly. All changes are visible as tracked insertions in the revised document. Below we address each point raised by Reviewer #1 in turn. Reviewer #2 comprised an automated ScreenIT report; we have reviewed all flagged items and addressed these separately below.

Reviewer #1

Comment 1: Sample restricted to healthy young adults

"By limiting the participants to healthy young adults, the research misses the chance to explore how these relationships manifest in older individuals or those with health conditions, who often struggle most with these simultaneous demands."

Response: We agree that this is a significant limitation. We included an explicit declaration in the Limitations section that the sample was limited to healthy young adults and that generalizability to older persons and clinical populations cannot be assumed. We have also included this for future research in the Implications and Future Directions section, explicitly urging for enrollment of older persons and clinical populations who experience the most functional consequences of impaired dual-task walking. We would like to clarify that the scope of the current study was deliberately limited to healthy young adults as a necessary first step in establishing baseline chronotype-gait associations before expanding to more complex populations where additional confounders such as medication, pathology, and age-related motor impairments would need to be addressed.

Change in manuscript: New tracked insertion in Limitations and expansion of Future Directions item (5).

Comment 2: Single afternoon session and time-of-day confound

"Conducting all assessments during a single afternoon session makes it difficult to ascertain whether the results truly stem from an individual’s internal rhythm or if they are merely a snapshot of mid-day energy levels. Without comparing these results against different times of day, such as early morning or late evening, the core argument regarding the influence of our biological clock remains somewhat incomplete."

Response: We completely agree that this is the most important drawback of the current study, and we addressed it openly in both the Discussion and Limitations sections of the original paper. In the revised version, we strengthened this acknowledgement with a tracked insertion in the Limitations which presently explicitly calls for within-participant repeated measurement designs assessing each participant at various times across the day to isolate chronotype trait effects from temporary time-of-day arousal fluctuations and directly test the synchrony hypothesis. We recognize that the current cross-sectional single-session design restricts causal inference, and we have presented all data as associations compatible with the synchronization hypothesis rather than verified circadian mechanisms throughout the text.

Change in manuscript: Tracked insertion expanding Limitation; comment marker added to Study Design section.

Comment 3: Absence of cognitive task accuracy data

"The primary focus on walking mechanics at the expense of mental performance. Without knowing how accurately the participants performed the mental tasks — whether they were solving the problems correctly or simply guessing — it is hard to determine if they were shifting their effort from thinking to walking."

Response: This is a valid methodological concern. The assessor verbally assessed cognitive task accuracy and reaction rates to ensure continued task engagement, but no formal scores were recorded on a trial-by-trial basis. In the revised manuscript, we added a tracked insertion in the Limitations section, emphasizing that without accuracy data, it is challenging to determine whether participants were successfully completing the cognitive tasks or randomly responding, which would conceal genuine cognitive-motor interference. We noted that the assessor constantly evaluated verbal engagement. during each trial, which gives proof that individuals were actively performing the tasks rather than disengaging completely.

Change in manuscript: Tracked insertion expanding Limitations; comment marker added to Dual-task conditions paragraph in Methods.

Comment 4: Laboratory setting and ecological validity

"The laboratory setting, while useful for control, lacks the unpredictability of a real-world environment where distractions and uneven surfaces are the norm."

Response: We agree. A new limitation paragraph has been added stating that it still needs to be determined if the observed chronotype-DTC associations would replicate under ecologically representative conditions. Mainly, we would like to point out that the controlled laboratory environment was a conscious design choice that allowed for exact spatiotemporal gait assessment using a three-dimensional motion capture device, which would not be possible in uncontrolled outdoor situations. However, we have expressly proposed the use of wearable inertial measurement devices deployed in realistic community settings as a viable next step, and ecological validity testing has been included under implications and future directions.

Change in manuscript: New tracked insertion as Limitation; Future Directions expanded with item (6).

Comment 5: Absence of data on daily variables

"The absence of data on daily variables like sleep quality from the night before, stress levels, or caffeine consumption leaves several external factors unaccounted for, all of which could significantly sway both mental agility and the rhythm of a person’s stride."

Response: We thank the reviewer for bringing this up. We would want to highlight that all participants' daytime drowsiness was assessed before to testing using the Epworth drowsiness Scale (ESS), a validated tool for detecting clinically relevant levels of daily sleepiness. Participants with ESS scores suggesting extreme daytime drowsiness were excluded from the research. This screening step immediately addresses the worry that poor prior-night sleep quality may have severely affected cognitive or motor performance on the day of testing, as participants whose sleep quality was poor enough to cause observable daytime drowsiness were excluded from the analysis.

We accept that the ESS measures habitual daytime drowsiness rather than acute same-day sleep quality, and that additional daily-state factors, such as morning coffee consumption and subjective stress levels on the testing day, were not systematically examined. These variables may impact both cognitive arousal and gait regularity, regardless of chronotype. We have included a paragraph in the limitations to acknowledge that and to state the need for future studies using structured day-of-testing questionnaires including, such as a single-night sleep quality item, a caffeine intake log, and a brief subjective stress rating, to separate acute daily-state effects from the stable chronotype trait. This recommendation is also listed under the future directions section.

Change in manuscript: Tracked insertion in Limitations; Future Directions expanded with item (7).

Reviewer #2 – ScreenIT Automated Report

Item 1: Randomisation not detected

"Not detected. If you performed an experimental study, please specify whether participants, animals or samples were randomly assigned to treatments or groups, and specify the randomisation procedure."

Response: this study is an observational cross-sectional study that does not involve an experimental protocol. The aim of this study was to evaluate the association between chronotype and dual-task cost on gait during various dual-tasks. Thus, randomization was not required. However, participants were recruited using convenience sampling.

Item 2: Blinding not detected

"Not detected. Please specify whether blinding was used at various phases of the experiment (e.g., blinding of patients, caregivers, outcome assessments, data analysis)."

Response: the following paragraph has been added in the manuscript to address blinding: considering the nature of the dual-task conditions, complete blinding of participants to the dedicated task was not applicable. However, to minimize demand characteristics, participants were not informed about the study hypotheses regarding the association between chronotype and gait performance. Moreover, the assessor who recorded gait data was not engaged in MEQ scoring or chronotype classification of participants, thus providing partial blinding for the primary outcome measure for assessors.

Item 3: Power analysis not detected

"Not detected. Please state how you determined the sample size for the study. If you performed a power or sample size calculation, please provide details."

Response: A new subsection titled “Sample size” has been added and the following paragraph has been added to address sample size determination: Sample size determination was performed using G*Power (version 3.1). Assuming a medium-to-large effect size (f² = 0.20) based on previous chronotype–cognition research, α = 0.05, power = 0.80, and five predictors (MEQ score, age, sex, BMI, and baseline gait performance), a minimum sample size of 55 participants was required.

Item 4: Open code

"If you wrote code to analyse your data, please consider sharing the code in a public repository. This makes it easier for others to reproduce your analyses and may also aid others seeking to analyse similar datasets."

Response: All statistical analyses were performed using IBM SPSS Statistics (version 25) via its graphical interface. No custom analysis code was written; therefore code sharing is not applicable to this study. All underlying data are publicly available on Zenodo (https://doi.org/10.5281/zenodo.17878983).

Item 5: Open data

"We found one or more references to open data in your manuscript that did not seem to be included in your data availability statement."

Response: The Zenodo link refers to our own primary dataset. The Data Availability statement in the revised manuscript has been expanded to explicitly confirm that all individual-level data underlying the findings, including spatiotemporal gait parameters, dual-task cost values, MEQ scores, ESS scores, SOMCT scores, and demographic variables, are publicly available at https://doi.org/10.5281/zenodo.17878983, with no data withheld.

Item 6: Conflict of interest statement

"It is important to supply a conflict-of-interest statement, even if there are none to declare, to transparently present the research."

Response: The authors declared no conflict of interest. A clear conflict of interest statement has been added at the end of the manuscript.

Item 7: Funding statement

"It is important to supply a funding statement because it makes the funding source and potential conflicts of interest transparent."

Response: This research received no external funding. A funding statement confirming this has been added to the revised manuscript.

Attachment Submitted filename: Response_to_Reviewers.docx

# Editor report: Decision Letter 1

**DOI:** 10.1371/journal.pone.0348387.r003

**Authors:** Julio Alejandro Henriques da Costa

16 Aug 2026

Association between chronotype and dual-task gait cost across distinct cognitive domains in healthy young adults

PONE-D-26-18506R1

Dear Dr. Kim,

We’re pleased to inform you that your manuscript has been judged scientifically suitable for publication and will be formally accepted for publication once it meets all outstanding technical requirements.

Within one week, you’ll receive an e-mail detailing the required amendments. When these have been addressed, you’ll receive a formal acceptance letter and your manuscript will be scheduled for publication.

An invoice will be generated when your article is formally accepted. Please note, if your institution has a publishing partnership with PLOS and your article meets the relevant criteria, all or part of your publication costs will be covered. Please make sure your user information is up-to-date by logging into Editorial Manager at Editorial Manager® and clicking the ‘Update My Information' link at the top of the page. For questions related to billing, please contact billing support.

If your institution or institutions have a press office, please notify them about your upcoming paper to help maximize its impact. If they’ll be preparing press materials, please inform our press team as soon as possible -- no later than 48 hours after receiving the formal acceptance. Your manuscript will remain under strict press embargo until 2 pm Eastern Time on the date of publication. For more information, please contact onepress@plos.org.

Kind regards,

Julio Alejandro Henriques Castro da Costa

Academic Editor

PLOS One

Additional Editor Comments (optional):

Reviewers' comments:

# Editor report: Acceptance letter

**DOI:** 10.1371/journal.pone.0348387.r004

**Authors:** Julio Alejandro Henriques da Costa

PONE-D-26-18506R1

PLOS One

Dear Dr. Kim,

I'm pleased to inform you that your manuscript has been deemed suitable for publication in PLOS One. Congratulations! Your manuscript is now being handed over to our production team.

At this stage, our production department will prepare your paper for publication. This includes ensuring the following:

* All references, tables, and figures are properly cited

* All relevant supporting information is included in the manuscript submission,

* There are no issues that prevent the paper from being properly typeset

You will receive further instructions from the production team, including instructions on how to review your proof when it is ready. Please keep in mind that we are working through a large volume of accepted articles, so please give us a few days to review your paper and let you know the next and final steps.

Lastly, if your institution or institutions have a press office, please let them know about your upcoming paper now to help maximize its impact. If they'll be preparing press materials, please inform our press team within the next 48 hours. Your manuscript will remain under strict press embargo until 2 pm Eastern Time on the date of publication. For more information, please contact onepress@plos.org.

You will receive an invoice from PLOS for your publication fee after your manuscript has reached the completed accept phase. If you receive an email requesting payment before acceptance or for any other service, this may be a phishing scheme. Learn how to identify phishing emails and protect your accounts at https://explore.plos.org/phishing.

If we can help with anything else, please email us at customercare@plos.org.

Thank you for submitting your work to PLOS One and supporting open access.

Kind regards,

PLOS ONE Editorial Office Staff

on behalf of

Dr. Julio Alejandro Henriques Castro da Costa

Academic Editor

PLOS One
